# Correction: Eocene intra-plate shortening responsible for the rise of a faunal pathway in the northeastern Caribbean realm

**DOI:** 10.1371/journal.pone.0249163

**Published:** 2021-03-22

**Authors:** Mélody Philippon, Jean-Jacques Cornée, Philippe Münch, Douwe J. J. van Hinsbergen, Marcelle BouDagher-Fadel, Lydie Gailler, Lydian M. Boschman, Fredéric Quillevere, Leny Montheil, Aurelien Gay, Jean Fredéric Lebrun, Serge Lallemand, Laurent Marivaux, Pierre-Olivier Antoine, with the GARANTI Team

In Fig 4, the geometry of cross-sections B and C is incorrect. Please see the correct Fig 4 here.

**Fig 4:**
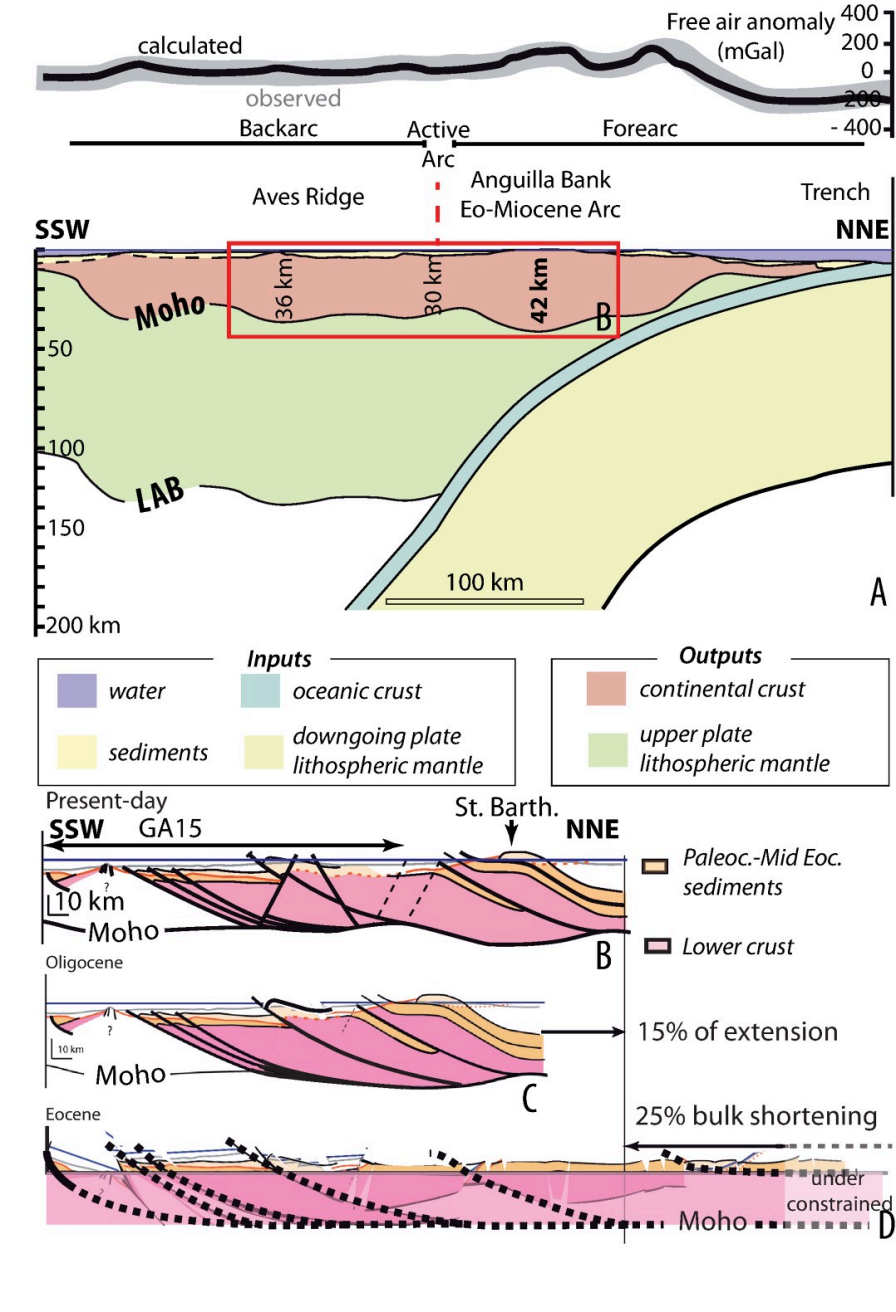
Crustal strain. A) Model of the crustal thickness (inversion of gravimetric data) along a NE-SW trending line from the trench to the Venezuela basin (location Fig 1C); B) Crustal strain pattern of the Northern Lesser Antilles; C) Restoration of the extensive deformation; D) Restoration of the compressive deformation.

## References

[1] Philippon M, Cornée J-J, Münch P, van Hinsbergen DJJ, BouDagher-Fadel M, Gailler L, et al. (2020) Eocene intra-plate shortening responsible for the rise of a faunal pathway in the northeastern Caribbean realm. PLoS ONE 15(10): e0241000. 10.1371/journal.pone.0241000 10.1371/journal.pone.0241000 33079958 PMC7575083

