# Supplementary material for: Eocene intra-plate shortening responsible for the rise of a faunal pathway in the northeastern Caribbean realm
Source: PLoS One. 2020 Oct 20;15(10):e0241000. doi: 10.1371/journal.pone.0241000 (PMC7575083; doi:10.1371/journal.pone.0241000)
Supplement: S1 Fig — Scale bars = 1mm. A) a) Coleiconus christianaensis (Robinson), b) Medocia sp., c) Textularia sp., sample PON8. B) Coleiconus christianaensis (Robinson), sample PON1. C) a) Halimeda sp., b) Praerhapydionina sp., c) Turborotalia pessagnoensis (Tourmakine and Bolli), sample PON4. D Planorotalites pseudoscitula (Glaessner), sample PON1. (DOCX) [file pone.0241000.s001.docx]

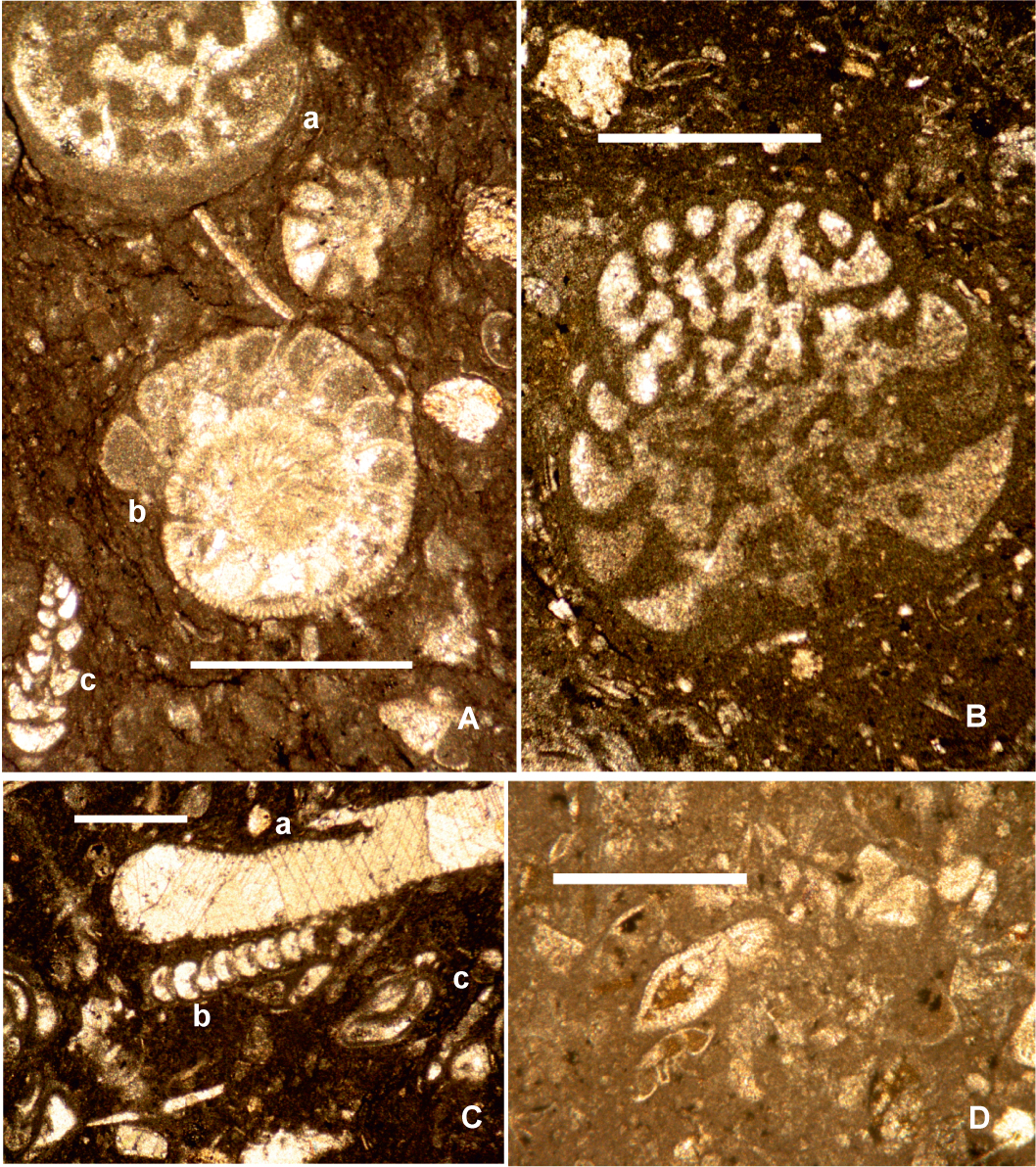


S1 Fig. Photomicrograph of the stratigraphical significant foraminifera found in Pointe Toiny limestones, Scale bars = 1mm. A) a) *Coleiconus christianaensis* Robinson, b) *Medocia* sp., c) *Textularia* sp., sample PON8. B) *Coleiconus christianaensis* Robinson, sample PON1. C) a) *Halimeda* sp., b) *Praerhapydionina* sp., c) *Turborotalia possagnoensis* (Tourmakine and Bolli), sample PON4. D *Planorotalites pseudoscitula* (Glaessner), sample PON1.
