# Supplementary material for: Eocene intra-plate shortening responsible for the rise of a faunal pathway in the northeastern Caribbean realm
Source: PLoS One. 2020 Oct 20;15(10):e0241000. doi: 10.1371/journal.pone.0241000 (PMC7575083; doi:10.1371/journal.pone.0241000)
Supplement: S1 File — (DOCX) [file pone.0241000.s002.docx]

S1 Appendix

**Geochronology: 40Ar/39Ar dating.**

Samples were crushed and sieved and a 100–200 μm grain size was retained for plagioclase separation. After magnetic separation, plagioclases were selected under a binocular microscope. The grains were leached with HNO3 (1N) for a few minutes and then repeatedly cleaned ultrasonically in distilled water and alcohol. Samples were packed in aluminum foil for irradiation in the core of the Triga Mark II nuclear reactor of Pavia (Italy) with several aliquots of the Taylor Creek sanidine standard (28.619 ± 0.034 Ma *in* [1]) as flux monitor. Argon isotopic interferences on K and Ca were determined by irradiation of KF and CaF_2_ pure salts from which the following correction factors were obtained: (^40^Ar/^39^Ar)K = 0.00969 ± 0.00038, (^38^Ar/^39^Ar)K = 0.01297 ± 0.00045, (^39^Ar/^37^Ar)Ca = 0.0007474 ± 0.000021 and (^36^Ar/^37^Ar)Ca = 0.000288 ± 0.000016. ^40^Ar/^39^Ar step-heating analyses were performed at Géosciences Montpellier (France). The gas extraction and purification line consist of (a) an IR-CO_2_ laser of 100 kHz used at 3-20% power to heat samples during 60 seconds, (b) a lenses system for beam focusing, (c) a steel chamber maintained at 10^-8^ - 10^-9^ bar, with a copper holder in which 2 mm-diameter blind holes were milled, and (d) two Zr-Al getters for purification of gases. Argon isotopes are analyzed with an Argus VI multi-collection mass spectrometer (with 4 faradays for masses ^40^Ar-^37^Ar and ion counting on ^36^Ar). One minute was allowed for equilibration before analysis. Mass discrimination of machines is followed daily. Mass discrimination was monitored daily using an automated air pipette and provided a mean value of 0.99985 ± 0.00274 per dalton. Single grain sanidine crystals were distributed in holes of the copper holder and were step heated. Blank analyses were performed every three sample analyses. Raw data of each step and blank were processed and ages were calculated using the ArArCALC-software [2]. Isotopic ratios were corrected for irradiation interferences and air contamination using a mean air value (^40^Ar/^36^Ar)_atm_ of 298.56 ± 0.31 [3,4]. Ages are statistically analyzed in two ways: ^39^Ar released spectra and inverse isochrones. Plateau ages are calculated from at least three consecutive ^39^Ar release steps comprising up to 50% of total ^39^Ar_K_ released and overlapping at the 2*σ* confidence level [5]. Isochrone ages are accepted when mean square weighted deviation (MSWD) is close to 1 and the ^40^Ar/^36^Ar intercept within 2σ from the (^40^Ar/^36^Ar)_atm_ value. The analytical data are reported in the **Table 1 to 4 and on Fig. 1**, and the errors are quoted at the 2σ level uncertainty including the error on the irradiation factor J.

**References**

1. Renne PR, Balco G, Ludwig KR, Mundil R, Min K. Response to the comment by W.H. Schwarz et al. on “Joint determination of 40K decay constants and 40Ar•/40K for the Fish Canyon sanidine standard, and improved accuracy for 40Ar/39Ar geochronology” by P.R. Renne et al. (2010). Geochimica et Cosmochimica Acta. 2011; 75: 5097–5100 .doi:10.1016/j.gca.2011.06.021
2. Koppers AAP. ArArCALCFsoftware for 40Ar/39Ar age calculations. *Computers & Geosciences*. 2002; **28**: 605–619.
3. Lee JY, Marti K, Severinghaus JP, Kawamura K, Yoo HS, Lee JB, Kim JS. A redetermination of the isotopic abundances of atmospheric Ar. *Geochimica et Cosmochimica Acta*. 2006; **70**: 4507–4512.
4. Renne PR, Cassata WS, Morgan LE. The isotopic composition of atmospheric argon and 40Ar/39Ar geochronology: time for a change. *Quaternary Geochronology*. 2009; **4**: 288-298.
5. Fleck RJ, Sutter JF, Elliot DH. Interpretation of discordant 40Ar/39Ar age- spectra of mesozoic tholeiites from Antarctica. *Geochimica et Cosmochimica Acta*. 1977, **41**: 15-32.

**
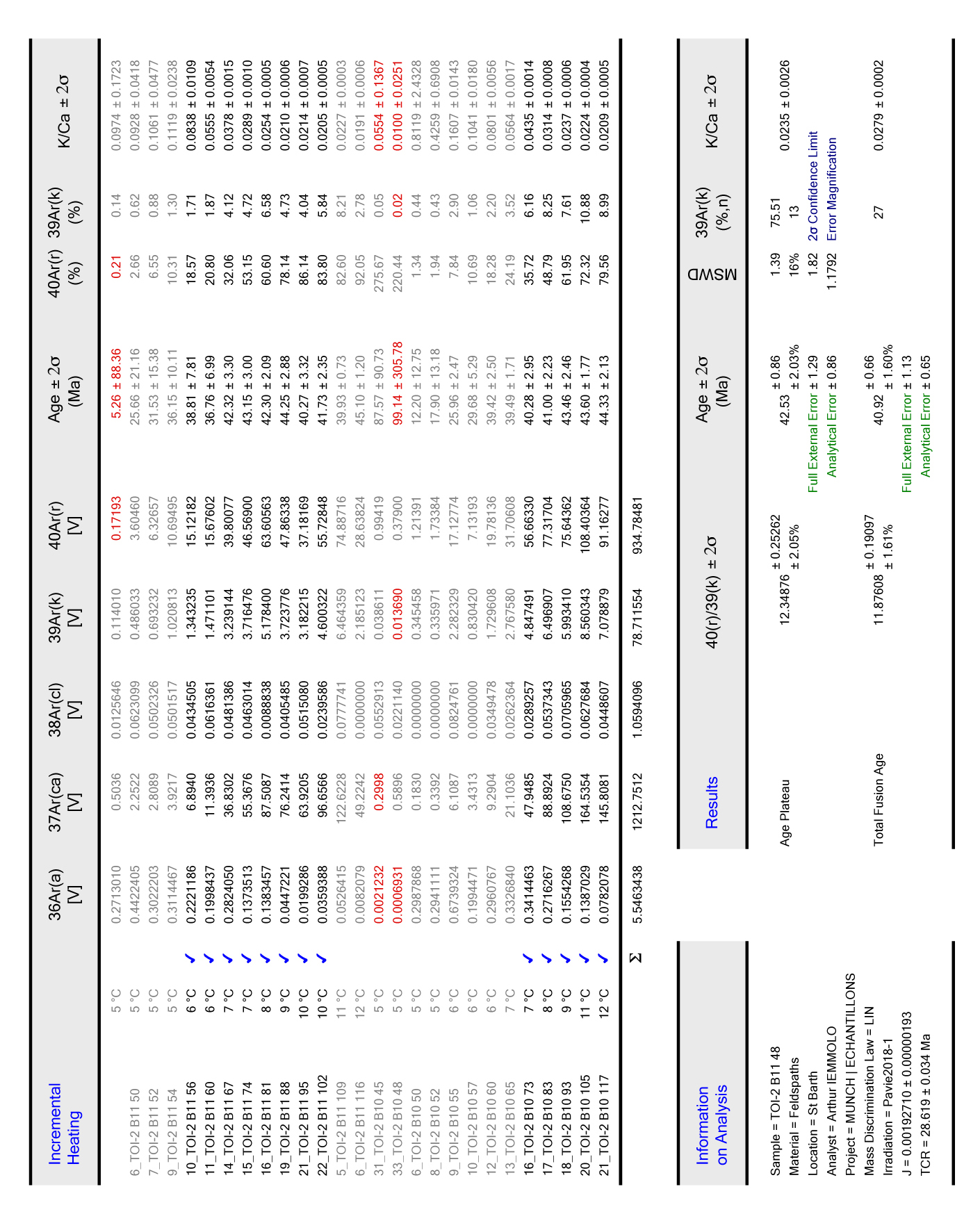
**

**Table 1.** Incremental Heating Summary.

**
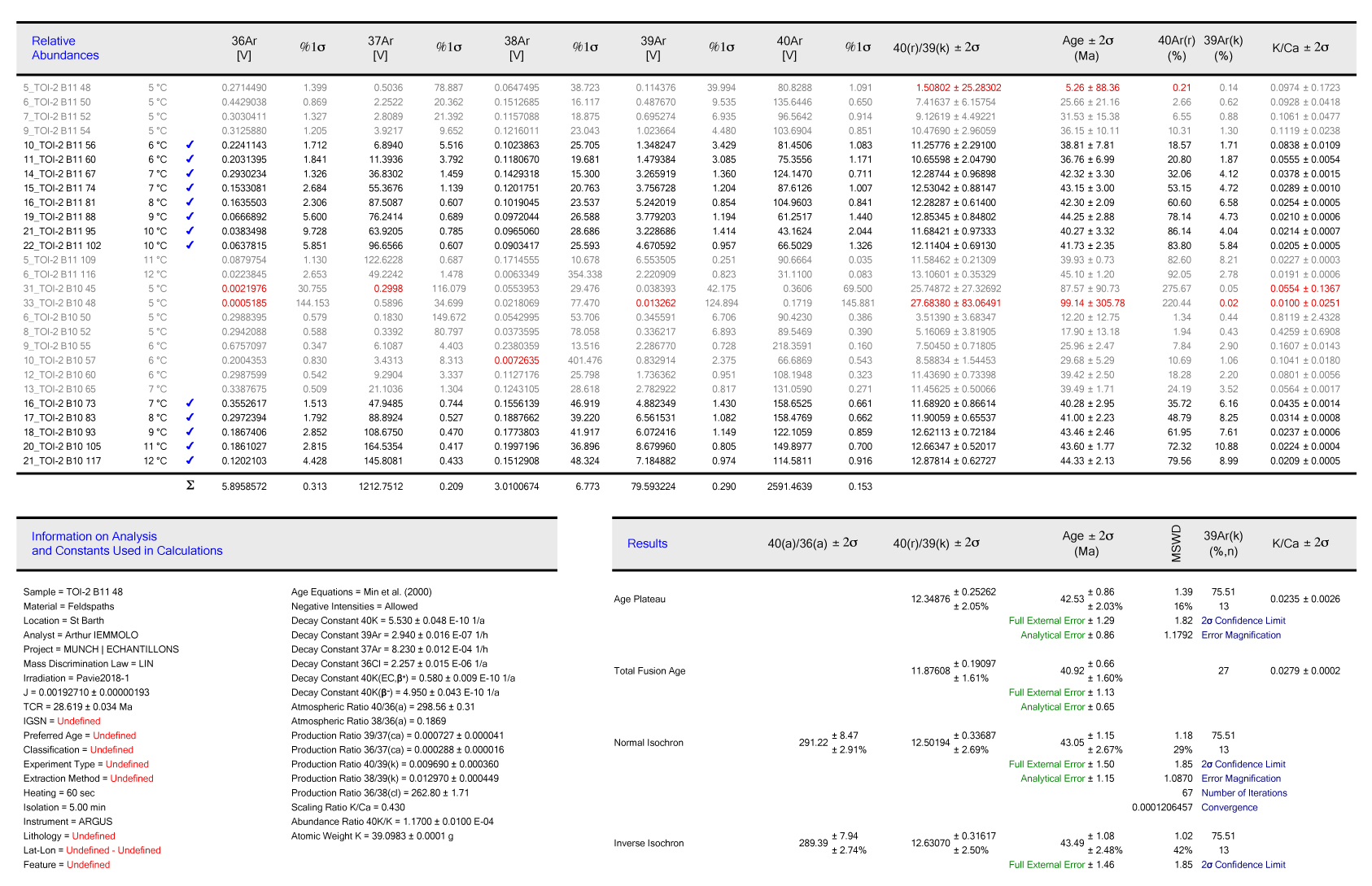
Table 2.** Relative Abundances.

**Table 3.** Sample Parameters.

**
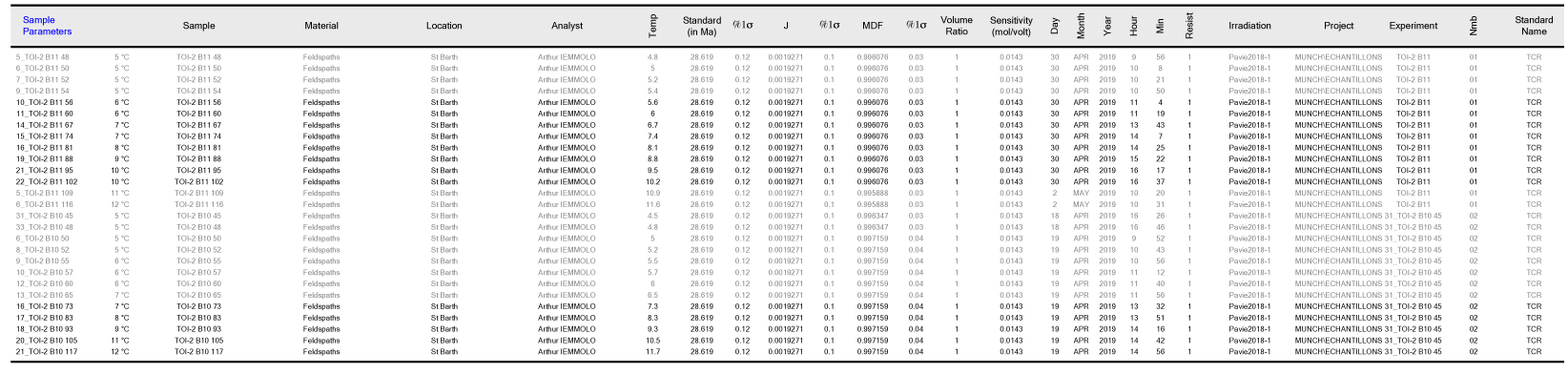
**

**
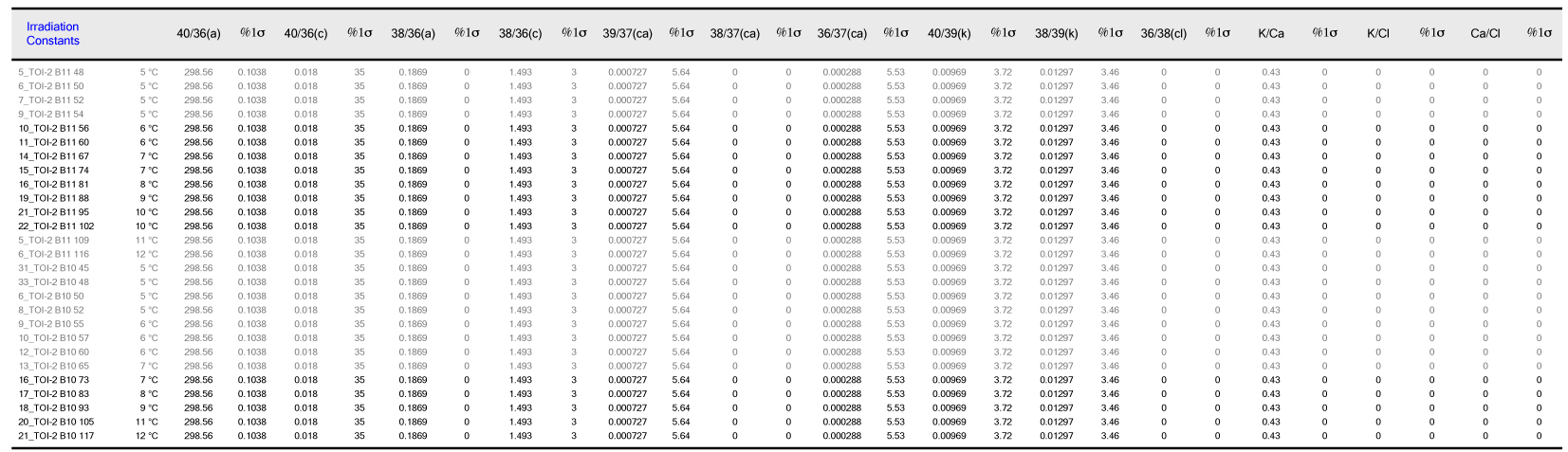
Table 4.** Irradiation Constants.

**
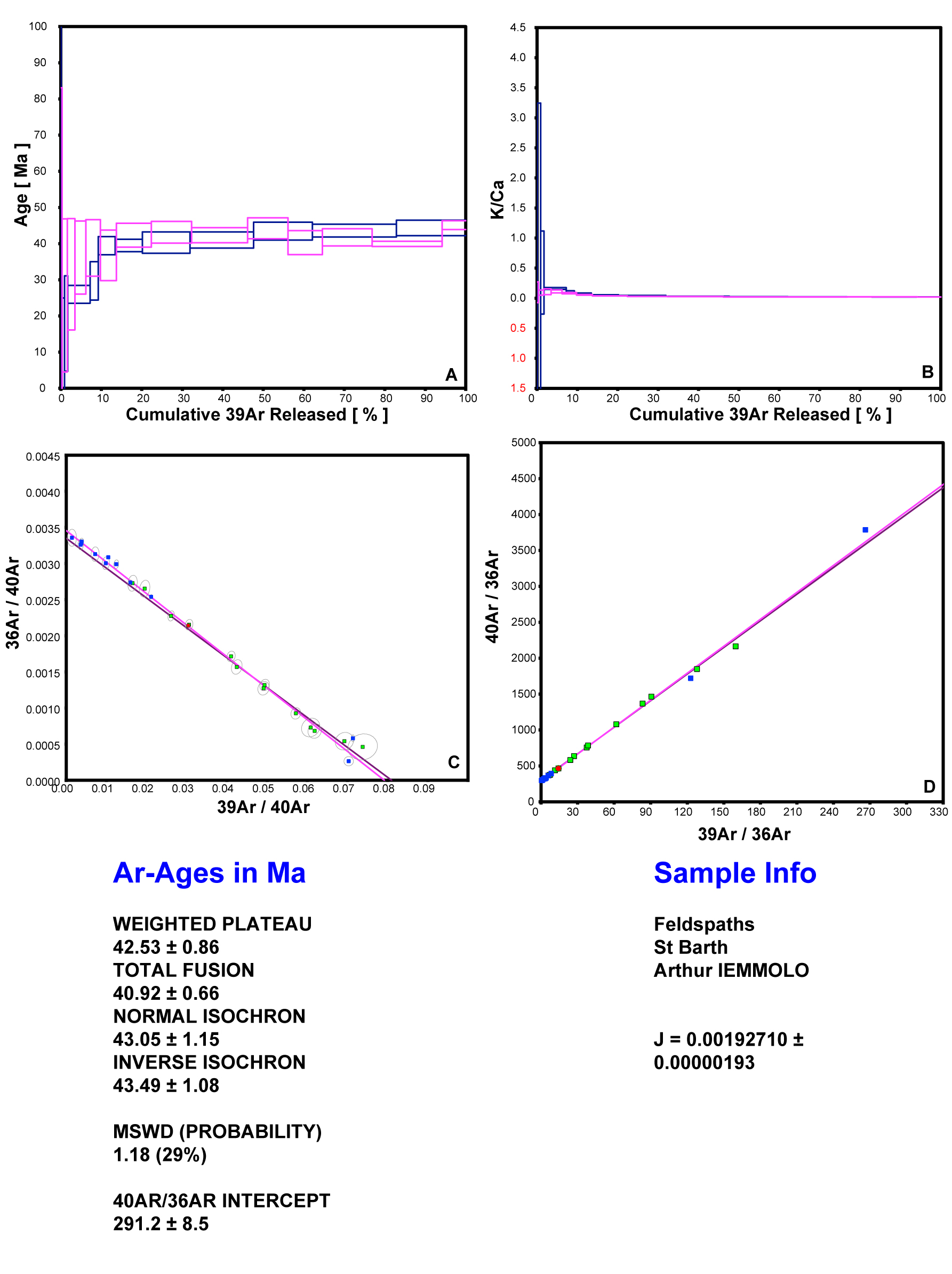
**

**Fig. 1.** Analytical results: A) Age plateau, B) K-Ca Plateau, C) Normal Isochron, D) Inverse Isochron.
