# Supplementary material for: Eocene intra-plate shortening responsible for the rise of a faunal pathway in the northeastern Caribbean realm
Source: PLoS One. 2020 Oct 20;15(10):e0241000. doi: 10.1371/journal.pone.0241000 (PMC7575083; doi:10.1371/journal.pone.0241000)
Supplement: S2 File — (DOCX) [file pone.0241000.s003.docx]

S2 Appendix

**Marine Geophysics: GARANTI cruise data acquisition and processing.**

During the survey GARANTI cruise, Mai 2017 ([1]; <https://doi.org/10.17600/17001200>), onboard the R/V L'Atalante, we recorded 41 multichannel seismic lines (MCS). We also interpreted multichannel seismic lines (MCS) acquired in the vicinity of the Anguilla Bank during ANTITHESIS [2-4]. Acquisition parameters for the selected MCS lines include a 3902 in^3^ airgun array source and a 720 traces - 6.25m trace spacing streamer ensuring a 120 fold coverage. Quality control and binning of the MCS data were performed on board using QCSispeed® and SolidQC® (Ifremer), and processing was performed using GEOCLUSTER® (CGG). Processing sequence includes band-pass (2–7–60–80 Hz) and FK filtering, Spherical divergence and amplitude (gain) correction, predictive deconvolution, 3 steps velocity analysis and Normal Move-Out (NMO) correction, external mutes, internal mutes and further multiple attenuation by 2D-Surface-Related Multiple Elimination and Radon domain filtering, velocity stack and constant velocity (1500m/s) FK migration. Our data set also includes petroleum seismic data courtesy of Saba Bank Ressources N.V. acquired in pseudo-3D over Saba Bank. Seismic data are described in [5].

**References**

1. JF Lebrun and S Lallemand (2017). <https://doi.org/10.17600/17001200>
2. B Marcaillou, F Klingelhoeffer, Antithesis Leg1 cruise, RV L'Atalante (2013) <https://doi.org/10.17600/13010070>
3. B Marcaillou, F Klingelhoeffer, Antithesis 3 cruise, RV Pourquoi pas ? (2016) <https://doi.org/10.17600/13010070>
4. JF Lebrun, B Marcaillou, Antithesis2 RECUP cruise, RV Antea (2015).<https://doi.org/10.17600/15005500>
5. AK Church, RE Allison, The petroleum potential of the saba bank area netherlands antilles. Search Discov. **10076**, 58 (2014).

**
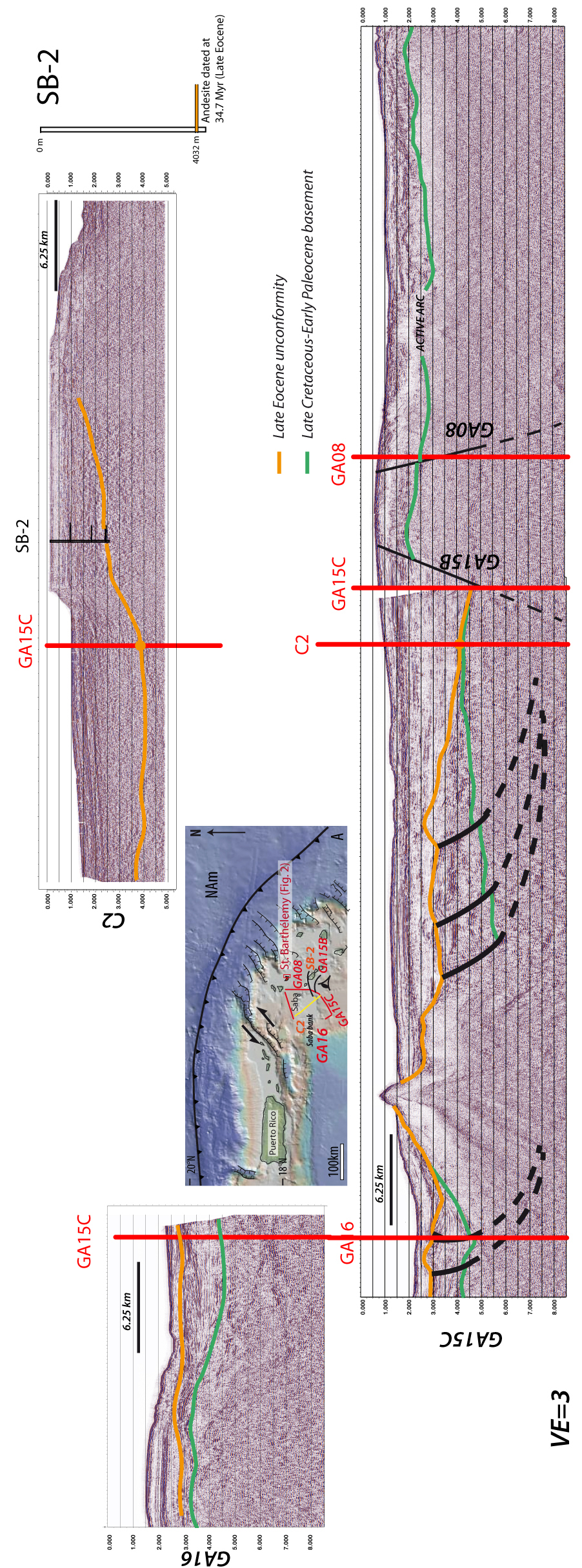
Fig. 1.** Map of the junction between the Greater and Lesser Antilles showing the location of the Oil industry lines C2 and the well SB-2 that have been correlated to the Garanti Line GA15C. The interpreted lines C2 and GA 08, 16, 15C and B, that are represented on the block diagram of Figure 3B, are also provided.
