# Supplementary material for: Eocene intra-plate shortening responsible for the rise of a faunal pathway in the northeastern Caribbean realm
Source: PLoS One. 2020 Oct 20;15(10):e0241000. doi: 10.1371/journal.pone.0241000 (PMC7575083; doi:10.1371/journal.pone.0241000)
Supplement: S3 File — (DOCX) [file pone.0241000.s004.docx]

S3 Appendix

**Gravity data modeling method.**

The figure 3C discussed in the main text presents a direct 2D gravity model of the crustal thickness using the free air anomaly data along a NE-SW trending line across the Northern Lesser Antilles forearc-arc -backarc region (**Fig. 1**). The free air anomaly is extracted from Bureau Gravimétrique International (BGI) data and products [1].

The 2D model was constructed using GMSYS software (Geosoft) in order to define the first order gravity structure. The model was extended at both extremities of the profile in order to avoid the edge effects. Regarding the large extent and great depth envisioned, 2D approach allows more realistic modeling with more geological and geophysical constraints that could be managed compared to 3D inversions [2]. The starting model was build using several constraints: slab contour from [3 and 4], sedimentary thickness from seismic dataset (Lines GA15 and GA08, Ant06, GARANTI and Antithesis cruise). The main gravity anomalies were accounted for by adding a continental crust layer which bottom was secondly inversed to better fit the observed curve. As mentioned by [5], Free Air anomalies are commonly suitable to investigate internal structures in submarine contexts. Large amplitude free-air anomalies are often reliable to large variation in the geometry of interfaces that separate layers with density contrasts.

Since seismic wave velocities (VP in km.s^-1^) and densities (kg.m^-3^) are two correlated parameters (various relationships are defined in the literature), density values for each layer were constrained using the empirical formula for the Nafe-Drake graphical formula published by [6]:

VP = 1.6612 ρ – 0.4721 ρ² + 0.0671 ρ^3^ – 0.0042 ρ^4^+ 0.000106 ρ^5^

The resulting densities values (**Table 1**) were also validated through a comparison with common values found in the literature.


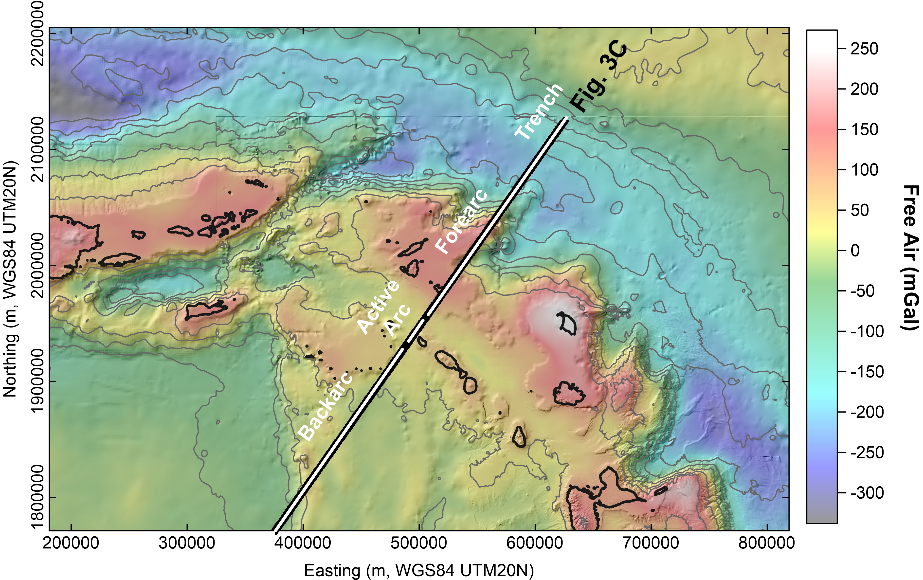


**Fig. 1.** Location of the gravity profile presented in figure 3C. The FreeAir data are extracted from BGI dataset (10).

**Table 1.** Equivalence between seismic wave velocities (VP in km.s^-1^) and densities (kg.m^-3^) for the main structures evidenced from seismic refraction profiles and geological observations.

**References**

1. BGI gravity data and products : Bureau Gravimetrique International (BGI). DOI:10.18168/BGI
2. LS Gailler, JF Lénat, 3D structure of the submarine flanks of La Réunion inferred from geophysical data. *Journal of Geophysical Reasearch*, **115**, B12, B12105 (2010). doi: 10.1029/2009JB007193.
3. M Laurencin, D Graindorge, F Klingelhoefer, B Marcaillou, M Evain, Influence of increasing convergence obliquity and shallow slab geometry onto tectonic deformation and seismogenic behavior along the Northern Lesser Antilles zone: *Earth and Planetary Science Letters*, **492**, 59-72 (2018). ISSN 0012-821X, <https://doi.org/10.1016/j.epsl.2018.03.048>.
4. Ó Gudmundsson, M Sambridge, A regionalized upper mantle (RUM) seismic model. *Journal of Geophysical Research: Solid Earth*, ***103*(B4)**, 7121-7136 (1999).
5. PJ Hart, The Earth's Crust and Upper Mantle. *Geophys. Monogr. Ser.*, **13**, AGU, Washington, DC. Publication Date: 00/1969 (1969).
6. WJ Ludwig, JE.Nafe, CL Drake, Seismic refraction, in The Se. A. E. Maxwell (Editor), **4**, *Wiley-Interscience*, NewYork, 53–84 (1970).
